# Supplementary material for: Damselfish face climate change: Impact of temperature and habitat structure on agonistic behavior
Source: PLoS One. 2020 Jun 30;15(6):e0235389. doi: 10.1371/journal.pone.0235389 (PMC7326182; doi:10.1371/journal.pone.0235389)
Supplement: Attachment — Submitted filename: rebuttal letter Plos One.docx [file pone.0235389.s002.docx]

**Rebuttal letter**

**Reviewer #1:** General comments:

**Dear reviewer #1, thankyou for your time and comments when revising our manuscript. In fact, the article approaches only one fish species, as it was a study developed during the master degree enrollment of the first author. Our aim was not to study the effects of warming on the whole ecosystem, as it would have taken several years, but however, we could address an up to date topic by responding a simple question of behavioral change, and thus we focused in the most characteristic behavior of an endemic fish species: aggression.**

**Regarding your concerns about the text, you will notice that we have accepted all suggestions from you and the other 2 reviewers, and the final version was modified and improved. The specific comment you have pointed are approached bellow:**

Specific comments:

Abstract:

Line 18: The author's statement “Due to the high anthropogenic impact of temperature, the pH, oxygen and structural complexity of many environments has changed.” sounds wrong. I would rephrase it. Warming is caused by the increase in atmospheric CO2 concentration, and the way that warming regulates pH could be somehow questionable, once that drops in pH (ocean acidification) is also directly associated with elevated atmospheric CO2 and primary production. **The first sentence was changed to be more adequate to the context of the article**

Line 24: This is unnecessary information “Fish behavior was recorded for 5 min before 25 and 5 min after mirror exposure.”- **sentence was removed**

Line 28-30: Motherhood statement that does not move the field forward and also does not match with the study finds. **Sentence was removed and a new final phrase was added**

Introduction:

**The introduction was revised and many parts were altered, as suggested.**

Line 35-58: The first two paragraphs could be easily mutated to a single paragraph. **Other reviewers have suggested changes to these paragraphs; thus it was almost completely changed.**

Line 80-91: The hypothesis tested is based on assumptions not evaluated during the experiment which in my opinion is the biggest flaw in the study. Climate change is known for affecting species with different strength (some species may have suffered stronger physiological setbacks than others), such negative effects could be diminished or buffered by environmental complexity (see Goldenberg et al. 2018) and species interactions. Additionally, behaviour changes could be a simple and straightforward strategy to diminish physiological impairments or even take a chance to boost performance (Ferreira et al. 2018). **The text was rewritten and the hypothesis is clearly presented.**

Materials and Methods:

Line 103: the sample size appears to be wrong by my calculations you collected 36 fish. Am I wrong? **We have included a statement presenting the reason why sample size differs. In fact, we have sampled 48 fish, however, due to sampling with a net, transportation to the lab and the changing in holding conditions, we have to exclude 12 fish. Some of them were excluded because of injuries during the sampling, other became sick at the lab (probably because these fish were not in the better health conditions when were captured and the stress may have declined their conditions, and other were excluded because they stopped feeding at the lab). Thus, final total number was 36 fish.**

Results:

First, due to low replication (sampling size vary from 6 to 12), I would appreciate seeing individual data points in the figures. **Changed as requested.**

Second, Figure 3, in my opinion, is not necessary. **We disagree** **that figure 3 should be deleted. It shows area occupancy before and after the mirror exposure and the figure indicates that fish were not using the closet area to the mirror before (Q4), but as soon as the mirror is shown, fish respond to it and occupied area Q1. Although it was not affected by temperature and complexity, the figure shows that fish still respond to the “intruder”.**

Figure 4, does not necessarily help since behaviour is not been affected by habitat complexity (left panel) and temperature (right panel) appears to have only a mild effect on fish behaviour, and as I said before, complexity buffer warming effects (Goldenberg et al. 2018). **Again, that figure shown behavioral change and as other reviewers did not mention anything related to excluding these figures, we decided to maintain them.**

I do have a question. During the behaviour test, the authors took feeding into account. However, is not clear in the experiment design how much food was available or how the authors made it available. Was it turf algae that grew in the experimental setup or it was food pallets? Was the food available in the same amount for all fish? Note that it could have serious implications on the results of the experiment. I would expect that fish in low complexity and higher temperatures would spend more time feeding (based on Metabolic Ecology Theory by Brown et al. 2004), or gardening (Ferreira et al. 2018, to avoid the overgrown of high nutritious algae by weedy ones in damselfish territories). Yet, the authors do not give any indication of such responses. **Indeed, we have not offered food during the behavioral test (10 min in the test tank). The dusky damselfish is known to be a gardener** **in the natural environment and the bottom nibbling is a innate behavior that was observed even when no food was available. Thus, we recorded the behavior as it was part of the repertory, but we changed the nomenclature to “substrate nibble” instead of “feeding”. However, we in fact observed that fish reduced feeding over the 30 days period, mainly in the barren warmer condition, but as we have not consistently recorded the amount of food consumed, we did not include this information in the MS.**

Discussion:

**The discussion was entirely revised and most of it re-written.**

Line 313-315: I haven’t seen any signal of plasticity or adaptability, the last not even close to being tested during the study. **Adaptability was removed from the sentence and we added a citation, as this information was not produced by our data, but is a result of other authors study**

Line 315-323: I could not understand the link between the previous sentence and this part of the text. **The sentence was removed and a novel paragraph was built**

Line 324-325: Strange sentence arrangement. Furthermore, the entire paragraph could be easily deleted. **As the text was re-written, the pointed phrase was removed**

I believe that the discussion needs to be rewritten and based only on the study finds, for example, what are the negative effects of being less aggressive? What is the physiological downside of change such behaviour? Could the behaviour change be beneficial to the individual, population or community? **We agree with the reviewer and the discussion was almost entirely re written.**

Jumping to the conclusion which I found extremely shallow, I would suggest the authors, to make deep changes and finalize the text with the most important take away message from the results. **The last part of the discussion is more robust in this new version.**

**Reviewer #2:**

**Dear reviewer #2, we are very glad with your comment and suggestions to improve our text. We have accepted all suggestions and you will find almost completely re-written text. We agree that we have been using a confusing statement when we say that *S. fuscus* is tolerant to thermic stress. In fact, these fish have more wide tolerance range, however, temperature changes affect them and cause lots of detrimental physiological and behavioral responses. We have changed our approach and included some novel statements to our MS.**

The questions for the manuscript are not clearly presented, thus it might be better to change the small summary at the end of the introduction (Lines 85-90) with the main research questions and predictions of what the authors expect to find. **We understand the point. We have changed the text and avoided characterizing the fish as thermotolerant, presenting the area and temperature range it occurs. From lines 85 to 90, it was completely rewritten and a clear hypothesis is presented.**

There is also information missing from some of the methods. The experimental design is a bit unclear, line 113 fore example, was there one fish per tank? Further there was no mention as to what was the temperature on the experimental tank where the behavior was recorded? was it 28C or 34C? This is important as some of the fish from the experiment might be responding to shock, rather than the experimental conditions. **Methods section was revised and we have included missing information pointed out by the reviewer. In fact, fish from each holding temperature was also tested in the same temperature, i. e. fish from 28 was tested in 28 degrees and fish from 34 was tested in 34 degrees. Also, fish were held in visual isolation from neighbours to avoid physical combat and injuries, but they were kept with chemical communication through water exchange.**

There is also no mention of the software packages used for the statistical analyses (was it all done in R?) **Univariate analyses (two-way and repeated measures ANOVA tests) were performed in the software Systat 12 and the multivariate procedures (PERMANOVA and SIMPER tests) in the software Primer 6 with PERMANOVA add-on. This information are now mentioned in the revised version of the manuscript.**

The discussion is unfortunately not very clear, as many ideas are repeated multiple times, making it difficult to follow. I must suggest the authors to revise this section extensively. The authors should mention in the discussion that the experimental temperatures are well within the range that these species experience today, and that conditions on those tidepools might be much warmer than 34C by the end of century.

Another limitation is that the authors don't discuss the specific effect of fish stress when being in a barren environment for along time. Please include in the discussion the manuscripts by:

- von Krogh, K., Sørensen, C., Nilsson, G. E., & Øverli, Ø. (2010). Forebrain cell proliferation, behavior, and physiology of zebrafish, Danio rerio, kept in enriched or barren environments. Physiology & behavior, 101(1), 32-39.

- Näslund, J., & Johnsson, J. I. (2016). Environmental enrichment for fish in captive environments: effects of physical structures and substrates. Fish and Fisheries, 17(1), 1-30.

There are some ideas in the discussion that are not well stated. For example, lines 359-361 suggest that changes in temperature lead to changes in salinity and O2, which leads to coral mortality. The main cause of bleaching is temperature increase and the loss of the association between symbionts and corals.

Finally, the authors could make some of the fish videos available on Youtube or other video sharing website, so colleagues can see the experiments. This would be a nice addition to the manuscript.**. We accept the suggestion and we have chosen one of the videos to make available on youtube. The address is cited on the MS.**

Minor revisions:

Line 39: The more drastic change in pH is caused by the increase of Partial pressure of atmospheric CO2. Thus I don't know how relevant pH is in this statement. **We removed mentions to pH**

Line 44: Underwater is one word **corrected**

Line 50: replace "species" with "organisms", since you are not talking about a specific group. **corrected**

Line 55-58: This line is a bit confusing, please consider reorganizing these ideas. **Re-written**

Line 59: I must encourage the authors to revise this. Coral reef fishes are very sensitive to changes in water temperature. Rephrase this to "Previous studies suggest that changes in behavior of coral reef fishes could be associated to fluctuations in environmental conditions". **Done**

Line 61: Replace "in this respect" with "Thus," **corrected**

Line 66: is this diel migrations, or actual long range movements? **We removed mentions to migration**

Line 67: Again I think it's tricky to suggest that some fishes are not affected by temperature. A much better approach could be just describing the distribution of the dusky damselfish, rather than speculating that it's not thermally sensitive. **corrected**

Line 81: "may increas 2-4C on average," **corrected**

Lines 85-90: This section should have the main questions of the manuscript. At the moment it is more like a summary of the paper. Please consider re-structuring this section. **Corrected according to studies from Dr. McCormick: In this study we evaluated whether water temperature and structural complexity of habitat may be related to changes in mobility patterns, tanks occupation and behavioral profile of *Stegastes fuscus*. For this, we subject the animals to classic mirror test and observe if animals kept at high temperature and barren conditions present significant differences in the mentioned variables when compared with group kept in high temperature and enriched habitat (similar to natural habitat). As an increase in temperature raises the metabolic rate of fish and this metabolic alteration promote direct influences in behavior of animals expected that high temperature groups and barren conditions show the lowest results.**

Line 104: replace "captures" with "samplings" **corrected**

Line 159: Please check the numbers of the Tanks. Is this the correct name of the treatments? **Names and numbers are correct**

Lines 313-315: I don't think adaptability was measured here, since the effect that was measured was plasticity. **ok, removed**

Lines 344-349: This section is confusing, as they both say very similar statements, but in one sentence it is temperature, but in the other it is CO2 and O2. Please edit this section for clarity. **It was re-written**

Lines 359: "such as salinity" **corrected**

Line 359-361: This doesn't seem right. Please edit for clarity. **corrected**

**Reviewer #3:**

**Dear reviewer #3, thankyou very much for considering our study and suggesting nice modifications to make it clearer and better. We are very thankful for your time and suggestions. We have accepted all of them and most parts of the MS (mainly introduction and discussion) were completely rewritten and new references were added. Please, find the new version of the MS with all suggestions from the 3 reviewers.**

General comments!

- The Dusky damselfish was considered by the authors as thermotolerant species, as being widespread along the Brazilian coast, as so, why this species would be a good indicator for changes in temperature or another impact related to climate change? If in the natural habitats, tide pools, this species can cope with 36oC, how to support this species as good to lab experiments on this topic instead of a less resistant species? **Two main reason made us focus on S. fuscus: 1 - Easy collection (high abundance) and (apparent) thermal plasticity; 2 - The species is known to tolerate high temperatures in tide pools for short periods of time (minutes / hours). The purpose of this paper is precisely to understand how animals would respond to long-term thermal stress (days), so it would not make sense to test this in another species that is known to tolerate short intervals of high temperatures**

- The hypotheses need to be clarified; the design is not clear for aggressiveness. **The experimental design was better explained in materials and methods. The hypothesis was more clearly described at the end of the introduction.**

- Damselfishes are very resistant to life in aquarium and definitely good for experiments in lab. Their natural diet includes algae and a little bit of live, animal material, depending on species. However, in captivity they can accept many different ratios. That said, if there is possibility of running experiments on the natural habitat, where different regimes of temperature can be managed, and fishes are health with a natural diet, why not have these comparisons done? **In fact, it would be excellent to compare data from nature to data from the laboratory. We plan to sample behavior in the field as we have**

**been observing fish behavior both in nature and in the lab. We observed several differences, for example in relation to the territorial defense, we observed that damselfish from nature are less aggressive against conspecifics than against heterospecific fish. However, we are still analysing these results and comparing the fish defence and neighbour recognition between field and lab sampling. We believe it will be ready for submission in 2020.**

- Two samples of twelve fishes (n=24) were reported, but disease events and discarded fishes were also mentioned, how many fishes were finally used to run each treatment and how was this managed with statistical analysis? How 36 experiments were run and only 24 fishes were available? It will be of great help see n=samples number above graphics!! **It is better explained in the MS so that the readers will see that we have sampled 48 fish, 12 held in each closed recirculating system. However, because sampling with a net, transporting and changing holding conditions are very stressful, some fish that we believe were not in its best health became sick or stopped eating and thus we decided to exclude them from the test. Thus, we ended up with 36 fish as described in the MS.**

- Each 12 tanks had the same circulation system, so there are two blocks missing in the analysis. **As I mentioned above, some fish were removed from the tests and tanks were maintained empty. We have included the details in the methods section, showing total number of animals used, number of animals composing each group and all statistical procedures.**

- What is a control for each treatment? **There is not a control for each treatment. We compared barren versus enriched, thus one is the control of the other. Also, 28 degrees is the usual temperature for maintain damselfish in the laboratory, thus it was the control condition for 34 degrees.**

- Q3 and Q4 seem to be same thing. It seems that from a 45 degrees of the mirror, if one are far from the base, it can see its reflection. **Yes, in fact it is the same, however, as using a single back area would double the size to be compared to the two front areas, we had to keep it separated into 2 areas.**

- How a fish was chose for the test tank? Are they take back for the same block and tank**? No, each fish was used only once and was part of only one group condition. Thus, each fish was recorded and then euthanized.**

- Did the Greenhouse-Geisser correction effect was tested anyway? **We used the Greenhouse-Geisser correction because the RM-ANOVA analysis returned epsilon values of 0.716 and 0.689. According to Girden (1992), when epsilon values are smaller than 0.75 the Greenhouse-Geisser correction is recommended.**

- I am not convinced that have time out of the analysis is corrected! Fish would count as a random factor. The Q comparisons seem not necessary. **Fish is the unit that is subjected to repeated measures both across time (before and after mirror) and on different quadrants (Q1, Q2, Q3, Q4), and therefore it is indeed a random factor. The comparison among quadrants was one of the main goals of our experimental design since the time spent in each quadrant reflect how aggressive the fish is (detailed explanation in Methods). The comparison between different times was only to evaluate eventual changes in basal behavior (before vs. after). To the best of our knowledge, there is no way of combining two levels of repeated measures in the same analyses, therefore we decided to separate it in two analyses, one for each time, using the repeated measures of the same fish on different quadrants as the response variable.**

- How the experiment has 96 degrees of freedom with only 36 replicates? **As each of the 36 fish was repeatedly measured four times, one in each of the four quadrants for each treatments combination, we end up with a total of 144 replicates and therefore 143 degrees of freedom (1 for each fixed factor (temperature and complexity) and their interaction (T x C); 32 for the residual of between subjects; 3 for the repeated factor (quadrants) and its interactions with fixed factors (Q x T; Q x C; Q x T x C); and 96 for the residual of within subjects.**

- The final message, “mitigating the effects of global warming", is tricky, but not of general interested for scientific public working in reef systems. For that, I suggest based on what the results show, to indicate further experiments needed to understand effects of temperature on reef organisms including fishes. Managers need to know how stressors will affect the normal functions of natural systems, especially those mediated by species. But also important is how they can implement management or conservation strategies that could mitigate global change effects. **We completely agree, thus we have changed our text to be clearer and to suggest next steps in this area of research.**

Line 40 – characterized by HIGH species diversity; **corrected**

Line 41 - Most of tropical reefs do not go through high temperature fluctuactions, so this generalization need to be rephrased; **corrected**

Line 42 – overtheir; **corrected**

Line 47 – For "feeding needs", you meant nutritional requeriments? **corrected**

Line 56 - Need rephrasing. Typical reported phase shifts events include domination of algae over corals. Corals are important framework of tropical reefs. Algae mortality is another interesting event but not usually reported for tropical reefs. Please, try to rephrase this sentence in order to a better understand of what process you really want readers to pay attention; **The text was rewritten based on suggestions. The part where we referred to algae and corals was removed.**

Line 65 – reproduction PATTERNS, COULD influence; **corrected**

Line 70 - This affirmative of no physiological responses in face of a high latitudinal distribution needs a reference support, it is just too speculative, but an interesting topic to be further investigated; **Unfortunately, there are no studies that relate the wide geographical distribution of the species in Brazil with physiological changes found throughout its distribution. On the speculation that the species is thermotolerant, we prefer to abide by the reviewers' suggestions and remove the part describing *S. fuscus* as a thermotolerant species.**

Line 75 – Need a reference in studies considering the Dusky Damselfish; **included**

Line 77 – Needs a proper reference; **As we did not find reference, we decided to remove the quote from “These species contribute to energy and nutrient transfer in reef environments, as a result of their gardening ability.”**

Line 84 – Energy=nutrients for damselfishes came from their turf matrix in territories, which includes algae, detritus and associated cryptofauna. All these itens can be part fo their diets pending on species. How these food sources will be affected by rise of temperature? Feeding rates clearly are affected by temperature. Territorial defence is a density dependant process which needs to be discussed considering the density of conspecifics and heterospecifics; **Although we will recognize that the food items of our study species will also be affected by future increases in water temperature and possible changes in the structural complexity of the reefs, the purpose of our study was limited to analyzing the behavioral aspect of the animal. During the experiments the animals were not fed and we did not quantify the nutrient intake by the animals. We agree that territorial defense is a process dependent on the density of co and heterospecifics. Therefore, in our experiment all animals were kept in social isolation both during the acclimatization / adaptation period and during the experiment period.**

Line 309 – One important aspect not evaluated by authors is feeding rates. Feeding rates is an important measure to understand patterns of energy flux throughout the food web. Lab experiments can also be important to test how feeding rates will be affected by temperature. Said that, the discussion need more on that issue; **we have included a discussion regarding feeding and methabolism**

Line 343 – Physiological displays = vigilance and immobility?? These are in fact behavioural aspects!! **corrected**

Line 359 – as salinity; **corrected**

Line 382 – How rise in water temperature may decrease food availability? This assumption need more details**. Increase in temperature may affect primary production and thus it can become a cascade that will end up reducing all the food chain. We have included a better approach to this topic in the discussion section.**
