# Supplementary material for: Damselfish face climate change: Impact of temperature and habitat structure on agonistic behavior
Source: PLoS One. 2020 Jun 30;15(6):e0235389. doi: 10.1371/journal.pone.0235389 (PMC7326182; doi:10.1371/journal.pone.0235389)
Supplement: Attachment — Submitted filename: Review PlosOne.docx [file pone.0235389.s003.docx]

Introduction

The authors have improved the MS with the previews reviews and I have few comments to clarify some points. I suggest the authors pointed out the effects of environmental changes (temperature and habitat) on reef fish behavior and what is its consequences for the coral environment. The authors should indicate the future forecast for the studied region, mainly the temperature and habitat structure projections. I suggest the authors rewrite the hypothesis since their hypothesis is only focused on temperature.

Matherial and Methods

It is not clear how did the authors calculate the swimming velocity through the Behavior recorded.

Minor Reviews

Line 38 – Change to “For water-breathing ectothermic …”

Line 323 – I suggest remove the discussion about aerobic scope since the authors did nor measured the fish metabolism and the discussion seems speculative.
