# Supplementary material for: Damselfish face climate change: Impact of temperature and habitat structure on agonistic behavior
Source: PLoS One. 2020 Jun 30;15(6):e0235389. doi: 10.1371/journal.pone.0235389 (PMC7326182; doi:10.1371/journal.pone.0235389)
Supplement: Attachment — Submitted filename: rebuttal letter 2 Plos One.docx [file pone.0235389.s004.docx]

**Rebuttal letter 2**

**Reviewer #2:** General comments:

**Dear reviewer, once again we thankyou for your comments on the MS. We have gone through your suggestions, which I comment below. The text was also completely revised for a native speaker.**

Specific comments:

line 37: the line of "ecological master factor" this sentence can be deleted **sentence was removed**
line 43: replace reef with reef's **Changed as requested.**
line 65: replace transpire with materialize **Changed as requested.**
Line 74-76: this is a very powerful conclusion that is not entirely supported by the data. I would suggest that the authors just say "we suggest that natural aggressive behavior of S. fuscus could be affected" **As the other referee also asked for changes in the last part of the introduction, you will find the text is changed and adequate to what you suggested**
Line 86: change ppm to ppt (parts per thousand in english). Check the entire manuscript as this is repeated inseveral places **Changed as requested.**
Line 104: this should be consistent throughout the manuscript, it should be 34C, not thirty-four degrees **Changed as requested.**
Lines 234-236: Please consider re-writing this line, it is a very important summary of the results, and it is a bit confusing at the moment **We have changed the paragraph to make it clearer**
Line 295: change ecological relation to "interactions" **Changed as requested.**
Line 296: the species "shows" **Changed as requested.**
Line 298: this has to be changed to " the rate of warming will accelerate in the near future" **Changed as requested.**
Line 311: change situation for "scenario" **Changed as requested.**
Line 323: you did not measure aerobic capacity or swimming speed so please consider re-shaping this section of the discussion. **In fact, aerobic capacity was not evaluated, however, we did measure swimming velocity, as present in figure 2. Thus, we have changed the text to adequate.**
Line 338: Include the abbreviation "C" for degrees Celsius. **Changed as requested.**
LIne 347-329: this line is totally out of context, you could eliminate it without affecting the manuscript. **Changed as requested.**
LIne 350-352: This section is confusing, please revise it. **The section was rewrite**
The authors could present a picture of the fish along with Figure 1. **We included the fish picture as suggested**
Figure 2 would be much better with Boxplots, this is just a suggestion and don't do the change if it's too much work. **The first draft sent the journal was a boxplot and other referee suggested changes because it was too polluted figure, thus, we decided not to change back**
Figure 2 and 3 need sub-headings A and B, to recognize what the bar graphs are representing. Check the journal formatting for this. **We have described graphs A and B in the legend, as you will see**
Check the format of the references, as some have different fonts, colors and styles. **Changed as requested.**

**Reviewer #4:**

**Dear reviewer #4, we are very glad about your comment to improve our text. We have accepted all suggestions, as some of them were also suggested by the other referee. We have changed our text according to your suggestions.**

**Introduction was changed in some parts to make the effects of environmental changes clearer and the hypothesis was rewritten to be more adequate.**

Matherial and Methods
It is not clear how did the authors calculate the swimming velocity through the Behavior recorded. **We have included a statement presenting how the software deals with the video to calculate velocity. The calculations are performed on a series of frames to produce quantified measurements of the animal behavior. It is known the position of the animals for each frame of the video, and the number of frames per second. Thus, the series of frames is analyzed, and the number of frames changed in a certain time is used to estimate the animal's movement.**

Minor Reviews
Line 38 – Change to “For water-breathing ectothermic …” **Changed as requested.**
Line 323 – I suggest remove the discussion about aerobic scope since the authors did nor measured the fish metabolism and the discussion seems speculative. **The aerobic scope was removed.**
